# Chaperone-mediated autophagy: the Achilles heel of the retinal pigment epithelium during age-related macular degeneration

**DOI:** 10.1080/15548627.2026.2636093

**Published:** 2026-03-04

**Authors:** Juan Ignacio Jiménez-Loygorri, Ana Maria Cuervo, Deborah A. Ferrington, Patricia Boya

**Affiliations:** a Department of Cellular and Molecular Biology, Centro de Investigaciones Biológicas Margarita Salas, CSIC, Madrid, Spain; b Tumour Biology Programme, Centro Nacional de Investigaciones Oncológicas (CNIO), Madrid, Spain; c Department of Developmental and Molecular Biology, Albert Einstein College of Medicine, Bronx, NY, USA; d Institute for Aging Research, Department of Medicine, Albert Einstein College of Medicine, Bronx, NY, USA; e Department of Medicine, Albert Einstein College of Medicine, Bronx, NY, USA; f Doheny Eye Institute, Pasadena, CA, USA; g Department of Ophthalmology, University of California, Los Angeles, CA, USA; h Department of Neuroscience and Movement Science, Faculty of Science and Medicine, University of Fribourg, Fribourg, Switzerland

**Keywords:** Age-related macular degeneration, chaperone-mediated autophagy, oxidative stress, proteostasis, retinal pigment epithelium

## Abstract

Chaperone-mediated autophagy (CMA) is a selective autophagy pathway that targets specific proteins containing a KFERQ-like motif for lysosomal degradation. It has been shown by us and others that CMA decreases during physiological aging in most tissues, and its impairment is associated with increased incidence of age-related pathologies, such as cardiovascular disease, neurodegenerative disorders or sarcopenia. However, its involvement in age-related macular degeneration (AMD), a prevalent progressive maculopathy that leads to bilateral central vision loss, had not been explored. In the early stages of AMD, the retinal pigment epithelium (RPE), a monolayer of cells that provides trophic support to photoreceptors, already presents major morphological and functional alterations but the cause of this cell type-specific vulnerability is unknown. In our latest work, we analyzed human donor RPE samples and found that CMA is selectively impaired in the RPE of AMD patients compared to healthy donors. These alterations lead to the accumulation of undegraded CMA substrates and untimely recycling of other proteins. Crucially, these findings are conserved in donor-derived iPSC-RPE models. We used this clinically relevant model to assess the consequences of dysfunctional CMA in AMD and found that it caused proteotoxicity, increased oxidative damage, and altered metabolism. Most importantly, using the new-generation CMA activator CA77.1, we restored proteostasis in AMD iPSC-RPE. Our findings shed light on the selective vulnerability of the RPE in AMD and provide evidence in support of CMA as a novel druggable target against AMD.

Mammalian autophagy encompasses three major pathways that differ in their substrates and lysosomal internalization route: macroautophagy, microautophagy and chaperone-mediated autophagy (CMA). While macroautophagy and microautophagy can also degrade organelles and lipids, among others, CMA selectively degrades proteins containing a KFERQ-like motif. KFERQ-like motifs arise from the combination of one or two positive residues (K, R), one or two hydrophobic residues (F,L,I,V) and one negative residue (E, D), flanked by a glutamine (Q) on either side. Furthermore, KFERQ-like motifs can also be generated by post-translational modifications (phosphorylation, acetylation) or be modulated by ubiquitination. Substrates are recognized by the chaperone HSPA8/HSC70 that delivers them to the lysosomal membrane by docking to LAMP2A. This event triggers the multimerization of LAMP2A, generating a translocation complex through which the substrate protein will enter the lysosome lumen, assisted by lysosomal HSPA8/HSC70. Acid hydrolases will break down the protein and the resulting amino acids will be shuttled back to the cytosol to fuel anabolic reactions. While CMA plays an important housekeeping role by eliminating damaged or superfluous proteins, it also acts as a master regulator of cellular processes such as glycolysis, lipid metabolism or the cell cycle, by degrading effector proteins involved in these processes in a timely and context-dependent manner.

Our group and others had previously described that macroautophagy is impaired in both the retinal pigment epithelium (RPE) and neuroretina during late stages of age-related macular degeneration (AMD), a maculopathy characterized by progressive bilateral central vision loss. Several genetic factors (e.g., CFH^Y402H^ [rs10490924] and ARMS2^A69S^ [rs1061170] variants) and environmental factors (e.g., smoking, diet) have been linked to increased risk of developing AMD. The RPE provides functional and trophic support to adjacent photoreceptors and, before any neuroretinal degeneration or histopathological hallmarks appear, presents metabolic and functional alterations in the early stages of AMD. We hypothesized that cell type-specific downregulation of CMA in the RPE could account for the selective primary degeneration of this tissue during early AMD and act as a turning point in the progression of the disease.

Using human publicly available single-cell and bulk RNA-seq data we identified a selective decrease of the CMA score, a weighted and directed average of expression of CMA-related genes that enables inference of the activation status of the pathway, in the RPE of AMD patients. Most importantly, no other cell type within the neuroretina-RPE-choroid interface showed similar alterations. We also performed a proteomic analysis of the RPE of donors with early AMD and observed an accumulation of proteins containing KFERQ-like motifs and an enrichment in validated CMA substrates. At the molecular level, we observed that the RPE of AMD patients presents decreased protein levels of both LAMP2A and HSPA8/HSC70, the key effectors of CMA.

To further dissect the consequences of impaired CMA on RPE degeneration during AMD, we generated iPSC-RPE cell lines from conjunctival cells of healthy and AMD donors. This model allowed us to study the contribution of the specific genetic makeup of each donor to CMA and proteostasis. Accordingly, we observed a similar decrease of the CMA score, reduced LAMP2A protein levels and increased proteotoxicity in iPSC-RPE of donors with AMD. To study the functionality of the pathway, we employed the KFERQ-Dendra reporter that monitors the lysosomal uptake of an exogenous CMA substrate. We confirmed that AMD donors present reduced CMA, although interestingly they are still able to activate the pathway in response to serum removal. CMA substrates are cell type-specific in order to respond to the specific needs of every tissue and its microenvironment. We analyzed the fraction of the proteome undergoing CMA by flux proteomics and identified shared and unique substrates in healthy and AMD iPSC-RPE. For example, healthy iPSC-RPE use CMA to degrade glycolytic enzymes (e.g. HK2 [hexokinase 2]) while AMD iPSC-RPE use it to recycle proteins involved in fatty acid metabolism and ferroptosis (e.g. SQLE [squalene epoxidase], or ACSL4 [acyl-CoA synthetase long chain family member 4]).

Recently, we developed first-in-class small molecules that selectively activate CMA by inhibiting a transcriptional repressor of the pathway. Among them, CA77.1 induces a robust activation of CMA across different cell types, presents a very favorable pharmacokinetic profile *in vivo* and can cross the blood-brain barrier and blood-retina barrier. We demonstrated that CA77.1 can induce CMA in iPSC-RPE and resolve proteostasis defects in cells from AMD donors. Most importantly, treatment with CA77.1 ameliorates mitochondrial dysfunction, improves NFE2L2/NRF2-mediated antioxidant response and reduces oxidative damage in the AMD cells (Figure 1).

**Figure 1.**
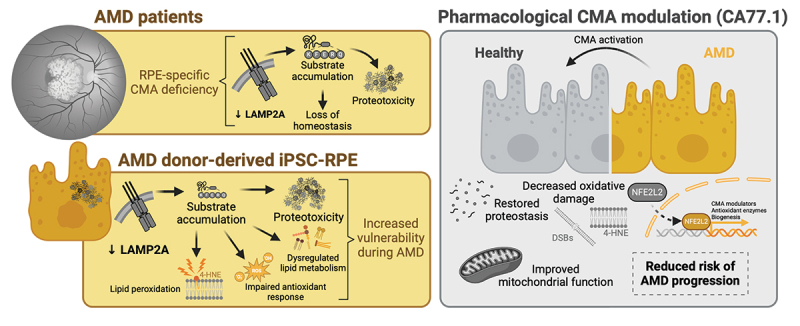
Selective chaperone-mediated autophagy (CMA) dysfunction in the retinal pigment epithelium (RPE) disturbs homeostasis and promotes age-related macular degeneration (AMD). Pharmacological upregulation of CMA in the RPE of AMD patients reverts proteotoxicity and improves metabolic output. Diagram created with BioRender.com.

In conclusion, we have identified CMA impairment as an RPE-specific alteration during the early stages of AMD and proposed it as a major driver of disease progression [1]. The use of human donor tissue and donor-derived iPSC-RPE cell lines, as well as the use of a novel and safe CMA activator (CA77.1), highlights the translational potential of our work. Furthermore, the contribution of CMA to RPE homeostasis warrants further exploration in the context of physiological aging and other diseases such as inherited Stargardt disease or certain types of retinitis pigmentosa.

## Data Availability

No original data is presented in the manuscript. Raw data from the reviewed manuscript is available in BioStudies (S-SCDT-10_1038-S44321-025-00329-w). Bulk and targeted proteomics iPSC-RPE data were deposited to ProteomeXchange (PXD051893). Additional information and resources are available from the corresponding authors.
